# Whole genome sequencing reveals great diversity of *Vibrio* spp in prawns at retail

**DOI:** 10.1099/mgen.0.000647

**Published:** 2021-09-28

**Authors:** Nicol Janecko, Samuel J. Bloomfield, Raphaëlle Palau, Alison E. Mather

**Affiliations:** ^1^​ Quadram Institute Bioscience, Norwich Research Park, Norwich NR4 7UQ, UK; ^2^​ Faculty of Medicine and Health Sciences, University of East Anglia, Norwich NR4 7TJ, UK

**Keywords:** comparative genomics, food safety, *Vibrio*, whole-genome sequencing

## Abstract

Consumption of prawns as a protein source has been on the rise worldwide with seafood identified as the predominant attributable source of human vibriosis. However, surveillance of non-cholera *Vibrio* is limited both in public health and in food. Using a population- and market share-weighted study design, 211 prawn samples were collected and cultured for *Vibrio* spp. Contamination was detected in 46 % of samples, and multiple diverse *Vibrio* isolates were obtained from 34 % of positive samples. Whole genome sequencing (WGS) and phylogenetic analysis illustrated a comprehensive view of *Vibrio* species diversity in prawns available at retail, with no known pathogenicity markers identified in *Vibrio parahaemolyticus* and *V. cholerae *. Antimicrobial resistance genes were found in 77 % of isolates, and 12 % carried genes conferring resistance to three or more drug classes. Resistance genes were found predominantly in *V. parahaemolyticus *, though multiple resistance genes were also identified in *V. cholerae* and *V. vulnificus *. This study highlights the large diversity in *Vibrio* derived from prawns at retail, even within a single sample. Although there was little evidence in this study that prawns are a major source of vibriosis in the UK, surveillance of non-cholera *Vibrio* is very limited. This study illustrates the value of expanding WGS surveillance efforts of non-cholera Vibrios in the food chain to identify critical control points for food safety through the production system and to determine the full extent of the public health impact.

## Data Summary

Epidemiological and genomic data for this project are available for all *Vibrio* genomes isolated from prawns in this study in the National Center for Biotechnology Information (NCBI) SRA database under accession PRJNA699735 and the supplementary material provided by this study.

**Impact Statement**

*
Vibrio* species are diverse bacteria predominantly found in estuarine and marine environments worldwide. Seafood products are an important vector associated with foodborne cases of vibriosis. This study evaluated prawns as a source of *Vibrio *, and cultured and sequenced 130 *Vibrio* spp from 211 prawn products sold at retail in the United Kingdom within a population- and market share-weighted survey. Through whole genome sequencing (WGS), a comprehensive evaluation of diversity of *Vibrio* species in prawns was conducted. Contamination of prawns was high, with *Vibrio* spp detected in 46 % of samples. WGS and phylogenetic analysis identified diversity of the *Vibrio* present within samples and demonstrated differences between prawn-derived and cases of human-derived *Vibrio* genomes, suggesting prawns are not a major source of vibriosis cases in the UK. Antimicrobial resistance genes were identified in 77 % of prawn-derived *Vibrio* genomes with 12 % of isolates containing resistance genes associated with multiple drug classes. This study has importance not only for creating a framework to direct efforts towards improving food safety throughout the food chain, but also heightens the understanding of microbial ecosystems as the high diversity within a single sample has critical implications for source attribution and outbreak investigations.

## Introduction

The true global burden of illness attributed to *Vibrio* exposure is underestimated and poorly understood due to the limited detection and surveillance of vibriosis [1–3]. *Vibrio* species are diverse Gram-negative halophilic bacteria found naturally in estuarine and marine environments worldwide with over 160 listed species currently identified [4]. In the food chain, *Vibrio* spp. are predominantly found in seafood products, consumption of which has been associated with human disease [5, 6]. In particular, *Vibrio parahaemolyticus, V. vulnificus, V. alginolyticus* and *V. cholerae* are important microbial hazards with characteristic virulence factors [7–9]. Not all virulence factors are well understood, however the presence of thermostable hemolysin genes (*tdh, trh*) in *V. parahaemolyticus* and *vvhA* hemolysin gene in *V. vulnificus* and the *ctxA* and *ctxB* toxin genes in *V. cholerae* are linked to and are utilised by public health laboratories to assess human pathogenicity [10–14]. Further characterisation of *V. vulnificus* ecotypes using genes *vcgC* (clinical), *vcgE* (environmental) and mutations within the *pilF* gene structure can inform the epidemiological lineage of presumably more virulent (clinical) and environmental strains [15–17]. The *tlh* gene in *V. parahaemolyticus* is a species-identifying virulence gene [10, 11]. *Vibrio* associated diseases manifest as mild to severe gastroenteritis, wound infections and in some cases septicaemia [6]. In the case of *Vibrio cholerae *, the O1 and O139 serotypes, which are the causative agents of pandemic cholera disease, are linked to contaminated water sources [18].

Prawn production is primarily established in marine waters of low and middle income countries (LMICs) with 67 % estimated to be farmed [19]. The production and consumption of prawns has increased substantially over the past 20 years worldwide [20, 21]. In the United Kingdom, 80 000 tonnes of prawns were imported in 2018, with a value of over £640 million [22]. Although not recognised as a major foodborne transmission route for *Vibrio* infections in humans, the intensification of production and increased consumption of prawns provides an opportunity for *Vibrio* to transmit through the global food supply chain [5, 8, 23, 24]. To combat viral and bacterial disease in production stocks, antimicrobials are commonly used for treatment and prevention. The reported usage of antimicrobials in this aquaculture sector varies between the indication of use, classes of antimicrobials chosen, and the volume used [19, 25–28]. Although surveillance data for antimicrobial use in prawn production is limited, use of these drugs in LMIC aquaculture sector is often unregulated, creating an aquatic reservoir of antimicrobial exposure [19, 29].

Antimicrobial resistance (AMR) has spread throughout the aquatic and surrounding terrestrial environment via clonal expansion of resistant bacteria and through horizontal gene transfer (HGT) of AMR genes [8, 19, 30]. Resistance to antimicrobial classes of critical importance to human medicine, including quinolones, higher generation cephalosporins and carbapenems, have been detected in seafood and the increase of multidrug resistant bacteria in aquatic ecosystems is a public health concern [5, 31–33].

Although *Vibrio* from a variety of ecological niches and along the food chain have been previously investigated [5, 8, 13, 14, 24, 30, 31, 34], these studies have typically focussed on specific *Vibrio* species and often do not include whole genome sequencing (WGS), which provides the highest molecular resolution to investigate bacterial evolution and population structure. One large cross-continental genomic investigation of *V. parahaemolyticus* identified human activity as responsible for changing the global distribution patterns of this organism, but the scope was limited to a single species [35]. To date little is known about the types and genomic diversity of *Vibrio* spp. found at retail, the implications to food safety and consequently to human health. The aims of this study were to (1) describe the prevalence of *Vibrio* in various prawn products at retail; (2) assess the sources, diversity, virulence potential and antimicrobial resistance genes of isolates; and (3) use this information to assess the potential human health impact of *Vibrio* in prawns and identify where interventions may most effectively be applied.

## Methods

### Sample collection

A population- and market share-weighted longitudinal study was used to collect prawn products at retail in Norfolk, United Kingdom. In brief, Office of National Statistics population estimates were used to define four strata of built-up areas by population size, and store market share data were used to classify stores as large (in the top ten food retail outlets) or small [36, 37]. Demographic and store market share percentages were used to generate the frequency and number of samples collected in each store type and stratum to ensure sampling reflected exposure by the general population of Norfolk (Table S1, available in the online version of this article). The raw and cooked prawns purchased included frozen, previously frozen or sold as fresh full body prawns, headless shell-on prawns, headless fully peeled prawns. No preference was given to prawn species, product origin or brand. All samples were packaged in zipper-lock bags to eliminate cross contamination and transported in coolers with ice packs and temperature data loggers. A cold chain was maintained during transit to the testing laboratory at Quadram Institute Bioscience, Norwich, UK.

### *Vibrio* detection and isolation

*Vibrio* was isolated from prawn samples using an adapted ISO 21872-1 : 2017 method [38]. Upon delivery to the laboratory, 100 g of each sample was placed into sterile filtered stomacher bags. Samples were homogenised (Seward stomacher 400C laboratory blender, Worthing, UK) in 225 ml of alkaline peptone water and incubated. All incubations throughout the isolation protocol were conducted at 28 °C for 18–24 h. Taking liquid from the top portion of the incubated broth, approximately 10 µl was inoculated onto thiosulfate citrate bile salt agar (TCBS) (Oxoid, Basingstoke, UK) and incubated. One presumptive yellow and one presumptive blue/green colony from each plate was further subcultured onto Columbia blood agar with 5 % sheep’s blood (Trafalgar Scientific, Leicester, UK) and incubated. In a subset of three prawn samples, six presumptive colonies (three yellow, three blue/green) per sample were selected and taken forward for further testing. Presumptive colonies were screened for oxidase production (Oxoid, Basingstoke, UK) and sensitivity to O129 discs was noted (Themo Fisher Scientific, Loughborough, UK). All oxidase-positive presumptive colonies were tested further and preserved in 1 ml of Brucella broth with 17.5 % glycerol in a biobank at −70 °C.

### DNA extraction and genome sequencing

DNA was extracted from each presumptive positive *Vibrio* isolate using the Maxwell RSC Cultured Cell DNA kit (Promega, Southampton, UK). Paired-end libraries were constructed using the Nextera XT DNA library preparation kit and libraries were sequenced as 150 bp paired-end reads on an Illumina NextSeq (Illumina, Inc., San Diego, CA, USA).

### Genomic analysis of short read data

Analyses were performed on the Cloud Infrastructure for Microbial Bioinformatics (CLIMB) server [39]. Raw reads were trimmed using Trimmomatic v0.36 [40]. Trimmed reads were assembled using Spades v3.11.1. The quality of the assemblies was assessed using QUAST v4.6.3, CheckM v1.1.2 and by aligning reads to the assemblies using the Burrows-Wheeler aligner v0.7.17 [41–43].

Assemblies were accepted if they consisted of less than 500 contigs that were over 500 bp, less than 50 duplicate genes and had a mean read depth of the four largest contigs above 30.

Prokka v1.13 was used to annotate assemblies [44]. Roary v3.13.0 was used to cluster annotated assemblies with a 95 % identity and core gene threshold and form a core gene alignment [45].

Speciation was analysed using Kraken2 Galaxy v2.0.7 [46]. ARIBA v2.14.1 was used to identify known plasmid types using the PlasmidFinder database, antimicrobial resistance (AMR) genes using the ResFinder database and virulence genes using the virulence finder database (VFDB) [47–50]. Resistance genes, virulence genes and plasmid groups were described by the gene cluster (as determined by ARIBA) with full gene nomenclature included in Table S2. For further analysis, AMR genes were grouped by antimicrobial class. A multiple drug resistance (MDR) gene profile was defined as an isolate containing genes conferring resistance to three or more antimicrobial drug classes [51]. Virulence gene detection was focused on known genes associated with clinical illness for *V. cholerae – ctxA, ctxB, V. parahaemolyticus – tdh, trh(X),* a related gene to *trh,* also referred to as *tdh-*related hemolysin*,* and *V. vulnificus – pilF, vcg, vvhA*. For *V. parahaemolyticus,* genus and family speciation gene *tlh* was included for *in silico* determination. For *V. vulnificus* genomes, the *vcgC* and *vcgE* alleles were investigated using a custom ARIBA database consisting of the *vcg* genes extracted from NZ_CP046835.1 and NZ_CP044069.1, respectively; and mutations in the *pilF* gene were investigated using a custom ARIBA database consisting of the gene from NZ_CP044069.1. ARIBA with databases downloaded from PubMLST (https://pubmlst.org/databases/) was used to determine sequence types of *Vibrio cholerae* and *Vibrio parahaemolyticus* genomes (Tables S3 and S4) [52–55].

Publicly available *Vibrio* genomes (*n*=127) were obtained to set the food survey genomes in context against a selection of clinical, food and environmental *Vibrio* genomes from EnteroBase *Vibrio* v.1.1.2 (https://enterobase.warwick.ac.uk/species/index/vibrio; accessed June 2019) [37], NCBI (https://www.ncbi.nlm.nih.gov/assembly/?term=vibrio) and published reference materials (Table S5). In the Enterobase database, context genomes were selected for each *Vibrio* species identified in the food survey. Selection criteria in Enterobase included collection years, sample source and location. Where possible, effort was made to match selection criteria to food survey genomes. Further screening was conducted on the ‘assembled’ column within Enterobase to select genomes matching the requested species.

To investigate the potential for transmission of *Vibrio* from prawns at retail to humans in the UK, a subset of clinically derived genomes in Enterobase, using collection year, lab contact equal to Public Health England (PHE) and keyword terms to specific to non-* V. cholerae* genomes was selected (*n*=52, accessed June 2019); this included *V. parahaemolyticus* (*n*=37), *V. cholerae* (*n*=7), as identified by assembled Enterobase genome results*, V. fluvialis* (*n*=4), *V. alginolyticus* (*n*=2)*, V. anguillarum* (*n*=1) and *V. antiquarius* (*n*=1). No clinical *V. vulnificus* genomes from PHE were available at the time of analysis. Where possible genomes were selected to match country or region of origin to food survey samples. Furthermore, *V. cholerae* genomes from each wave of the seventh cholera pandemic were included from published sources as additional comparators of *V. cholerae* genomes detected in this food survey (Table S5).

### Phylogenetic analysis

RaxML v8.2.4 was used to generate a maximum-likelihood tree based on single nucleotide polymorphisms (SNPs) in the core gene alignment [56]. The process was repeated with *Photobacterium damselae* (SAMN06909323) to root the tree.

For *V. cholerae, V. parahaemolyticus* and *V. vulnificus* genomes, Snippy v3.2 (https://github.com/tseemann/snippy) was used to align reads to the reference genomes (* V. parahaemolyticus* (SAMD00058707), *V. cholerae* (SAMN02603969) and *V. vulnificus* (SAMN04457466) (Table S5). Gubbins v2.3.1 was used to remove SNPs putatively associated with recombination [57]. The processes were repeated with a *V. parahaemolyticus* reference (SAMD00058707) for *V. cholerae* and a *V. cholerae* reference (SAMN02603969) for both *V. parahaemolyticus* and *V. vulnificus* to root the trees. Tree visualisation and annotation were achieved with the Interactive Tree of Life (iTOL) version 4.4.2 (https://itol.embl.de/). TreeCluster v1.0.1 was used to identify clusters of *V. cholerae* and *V. parahaemolyticus* isolates using the maximum-likelihood trees [58].

### Data availability

Epidemiological data and accession numbers for all *Vibrio* genomes isolated from prawns in this study were submitted to the National Center for Biotechnology Information (NCBI) SRA database under accession PRJNA699735 and are available in Table S6.

### Statistical analyses

Metadata descriptive analysis and logistic regression were performed using SAS version 9.4 (Cary, NC, USA). A sample was considered positive if one or more *Vibrio* isolates confirmed by sequence predicted taxonomic identification (Kraken2) were detected. These results were analysed to identify if there was a significant difference in the proportion of samples positive by specific sample type. This analysis was conducted in Open Source Epidemiologic Statistics for Public Health OpenEpi Version 3.01 (www.OpenEpi.com). Confidence intervals (two tailed 95 %) were calculated around prevalence estimates.

Univariable logistic regression models were used to identify factors associated with *Vibrio* spp. positive samples; candidate explanatory variables included sample type (raw, cooked, full body, headless with shell on, headless peeled), country of origin and region of origin. Regions of origin variable was created due to the low number of samples in some countries of origin.

## Results

### Sample demographics, *Vibrio* prevalence

Between May 2018 to April 2019, 211 prawn samples were collected from retail outlets over 42 sampling trips (Table S1). Retailers included 202 large chain stores and nine small independent grocers or fishmongers. Collected prawn samples were categorised into raw or cooked, then further divided into six subtypes (86 raw headless peeled, 33 raw full body, 30 raw headless shell on, 48 cooked peeled, eight cooked shell on, six cooked full body (Fig. S1)). *Vibrio* spp. were identified in 97 (45.9 %) of the samples tested with a total of 130 *Vibrio* isolates further analysed. The proportion of samples testing positive for *Vibrio* was highest in raw full body prawns (81.8 %), followed by raw headless shell on (70.0 %) and raw headless peeled (51.2 %) (Table 1). Of the 62 cooked prawn samples collected, five samples (8.1 %) contained *Vibrio *.

**Table 1.** Description and prevalence of *Vibrio* species by prawn sample sub-categories collected from 211 prawn samples at retail

	no. (%)					
Prawns (number of samples)	Prawn raw full body	Prawns raw headless peeled	Prawns raw headless shell on	Prawns cooked full body	Prawns cooked peeled	Prawns cooked shell on
Number of samples tested (*n*=211)	33 (15)	86 (41)	30 (14)	6 (3)	48 (23)	8 (4)
Number of samples positive (*n*=97)	27 (28)	44 (45)	21 (22)	2 (2)	2 (2)	1 (1)
Number of *Vibrio* isolates collected (*n*=130)	35 (27)	58 (45)	31 (16)	3 (2)	2 (2)	1 (1)
*** Vibrio* species (number of isolates**)						
***V. parahaemolyticus (n=83)***	20 (24)	41 (48)	20 (24)	2 (2)	0 (0)	0 (0)
***V. cholerae (n=5)***	3 (60)	2 (40)	0 (0)	0 (0)	0 (0)	0 (0)
***V. alginolyticus (n=4)***	1 (25)	1 (25)	2 (50)	0 (0)	0 (0)	0 (0)
***V. vulnificus (n=3)***	0 (0)	1 (33)	2 (67)	0 (0)	0 (0)	0 (0)
*** Vibrio* spp. (other) (*n*=35**)	9 (27)	14 (42)	9 (27)	1 (3)	2 (6)	1 (3)
*Vibrio owensii (n=9)*	4 (44)	2 (22)	3 (33)	0 (0)	0 (0)	0 (0)
*Vibrio campbellii (n=7)*	1 (14)	5 (71)	1 (14)	0 (0)	0 (0)	0 (0)
*Vibrio diabolicus (n=6)*	1 (17)	1 (17)	0 (0)	1 (17)	2 (33)	1 (17)
*Vibrio tubiashii (n=6)*	2 (33)	2 (33)	2 (3)	0 (0)	0 (0)	0 (0)
*Vibrio anguillarum (n=3)*	1 (33)	2 (67)	0 (0)	0 (0)	0 (0)	0 (0)
*Vibrio harveyi (n=1)*	0 (0)	1 (100)	0 (0)	0 (0)	0 (0)	0 (0)
*Vibrio mimicus (n=1)*	0 (0)	0 (0)	1 (100)	0 (0)	0 (0)	0 (0)
* Vibrio* novel-1 *(n=2)*	2 (100)	0 (0)	0 (0)	0 (0)	0 (0)	0 (0)

*Vibrio parahaemolyticus* accounted for 64 % (83/130) of all *Vibrio* identified and was isolated from 31 % (66/211) of all prawn samples tested, with raw sample types containing the majority of *V. parahaemolyticus* (Table 1). *V. parahaemolyticus* was detected from all geographical regions represented in purchased prawns (Table S7), although region was not significantly associated with the detection of *V. parahaemolyticus* (Table S8).

Non-toxigenic *Vibrio cholerae* (non-O1/O139), *V. alginolyticus* and *V. vulnificus* were found in 3.8, 3.1 and 2.3 % of samples, respectively, and only in raw prawn products. Other *Vibrio* species accounted for 27.7 % of the overall prevalence (Table 1). Contamination of prawn samples with *Vibrio* differed among sample subtypes (Table 1). Raw prawns were significantly more likely to test positive for *Vibrio* than cooked prawns (OR 18.15; CI 7.27, 53.93; *P*<0.0001) and raw full body prawns were significantly more likely to test positive for *Vibrio* than raw peeled prawns (OR 4.25; CI 1.64, 12.31; *P*<0.05).

### Phylogenetic analysis

**Fig. 1.**
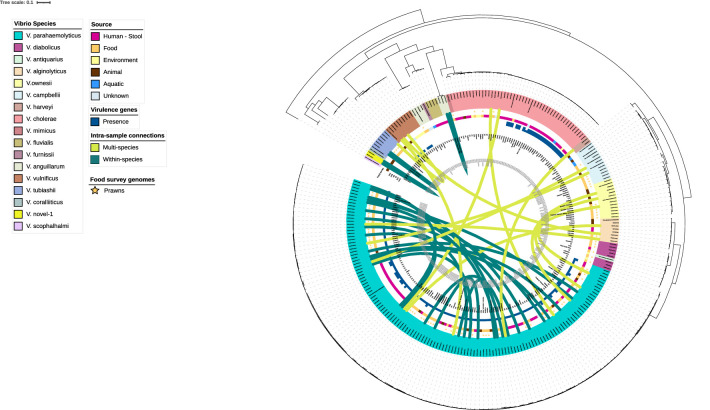
Maximum-likelihood phylogeny using core gene alignment of 130 *Vibrio* isolates collected from prawns sold at retail and 127 context genomes of clinical, environmental and food origin. *Vibrio* species are indicated by the colour of the tree tips. Ring from outer to inner (**a**) food survey sample genomes - star; (**b**) sample source; (**c**) virulence gene presence; (**d**) country of origin labels; (**e**) date of collection. Dark green arrows indicate within *Vibrio* species inter-sample connection; light green arrows indicate multiple species inter-sample connection.

The phylogenetic relationships of the 130 *Vibrio* spp. genomes from the 211 prawn samples in this study along with the context collection of publicly available genomes from human clinical, environmental and other food samples are shown in Fig. 1. Of the 97 prawn samples with at least one *Vibrio* isolate identified, 66.0 % (*n*=64) contained one speciated *Vibrio* isolate, 20.6 % (*n*=20) contained two *Vibrio* isolates of the same species, and 13.4 % (*n*=13) contained two different *Vibrio* species. Of the twenty samples with two *Vibrio* isolates of the same species, seventeen had two *V. parahaemolyticus *, one had two *V. anguillarum *, one had two *V. tubiashii* and one had two previously undefined *Vibrio* spp. hereafter called *Vibrio* novel-1.

**Fig. 2.**
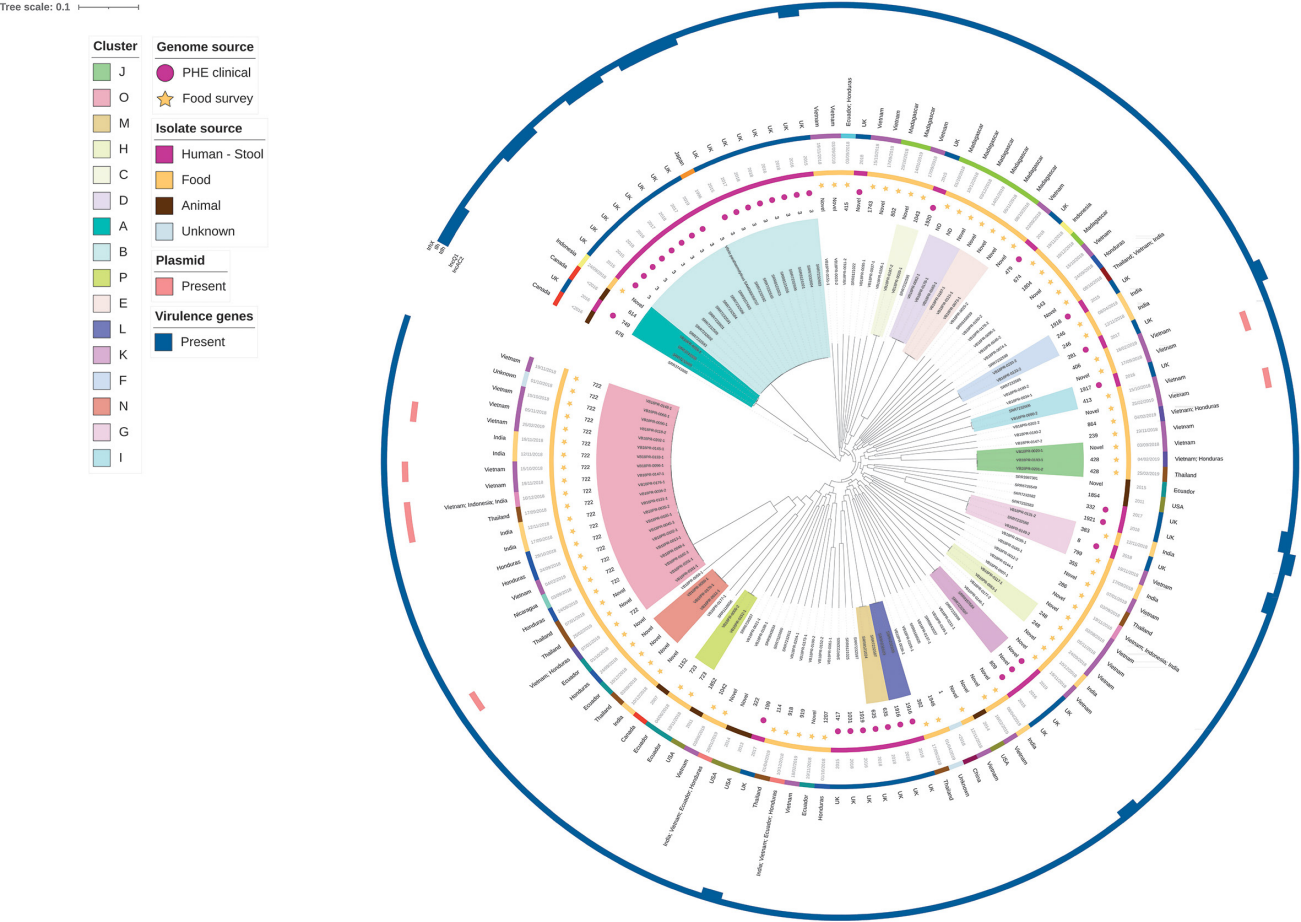
Maximum-likelihood phylogeny using core genome alignment of 83 *V. parahaemolyticus* isolates collected from prawns sold at retail and 48 context genomes from clinical, environmental and food sources at collected in different years and geographical regions. Phylogenetic clusters (*n*=16) are indicated in shaded areas of tip labels. From the inner ring (**a**) sequence types; (**b**) project code indicated by star (food survey) and circle (Public Health England clinical); (**c**) sample source; (**d**) date of collection; (**e**) country of origin; (**f**) plasmid presence; (**g**) virulence gene presence.

The species-specific analyses provide higher resolution data to compare the between- and within-sample diversity of samples containing isolates of the same species. The population of *V. parahaemolyticus* analysed (food survey and contextual genomes) was diverse with a pairwise non-recombinant SNP difference range of 0–2017 (median 1740) and 27 known STs, 32 novel STs and two undetermined STs (Fig. 2). The phylogenetic tree is marked by many deep branches, indicating highly divergent genomes, with a small number of clades representing more closely related genomes. There were 16 clades containing genomes with pairwise non-recombinant SNP differences ranging from 0 to 26 (Table S10). The smallest cluster size contained two genomes while the largest contained 21 genomes (Fig. 2). Food survey derived *V. parahaemolyticus* genomes were included in 12 clusters, with three clusters including both food survey and clinical isolates from Public Health England (PHE) (Table S10). In cluster A, consisting of three genomes, there was a 26 pairwise non-recombinant SNP difference between a prawn derived *V. parahaemolyticus* genome collected in 2018 and the nearest clinical derived genome collected in 2016 in the UK (Table S11). While the clinical derived genome in cluster A contained the virulence gene *tdh*, the prawn derived genome did not; in addition, the two genomes were of different STs and had different plasmid and resistance gene profiles. In clusters G and I, no similarities in ST, plasmid, AMR genes or virulence profiles were found. Clinical PHE genomes were collected in 2016 and early 2018, while prawn derived genomes were collected in late 2018. Differences in sequence types, virulence gene profiles or resistance gene profiles were observed in most clusters; the most similar cluster was cluster O, the largest with 21 genomes. All 21 genomes in this cluster derived from prawns, none were from human clinical cases. The cluster O pairwise non-recombinant SNP differences ranged from 0 to 18 and included 19 ST722 genomes and two novel sequence types. Two novel STs in cluster O were three non-recombinant SNPs apart, both originating from king prawns harvested in Thailand. Within cluster O, all prawn samples were king prawns harvested from seven different locations from as far apart as Vietnam and Honduras, with one unidentified location.

The species marker gene *tlh* was exclusively identified in *V. parahaemolyticus* genomes. Pathogenicity indicator genes *tdh* and *trh* were not identified in any food survey prawn derived *V. parahaemolyticus* genomes but were also missing from most PHE clinical isolates (Fig. 2). Plasmid group *Inc*A/C2 was detected in 2/83 genomes and *Inc*Q detected in 5/83 *V. parahaemolyticus* genomes using the PlasmidFinder database (Fig. 2).

Two out of the three *V. vulnificus* isolates collected from food samples had a *vcgC* (clinical) allele of the *vcg* gene (Fig. 2) [17]. The *vcg* gene from the remaining food *V. vulnificus* isolates shared less than 95 % homology with the *vcgC* or *vcgE* alleles investigated. All three *V. vulnificus* isolates collected from prawns contained the *vvhA* gene and I179V, V187I and A189T mutations in the *pilF* gene associated with isolates derived from clinical infections [16].

### V. cholerae

**Fig. 3.**
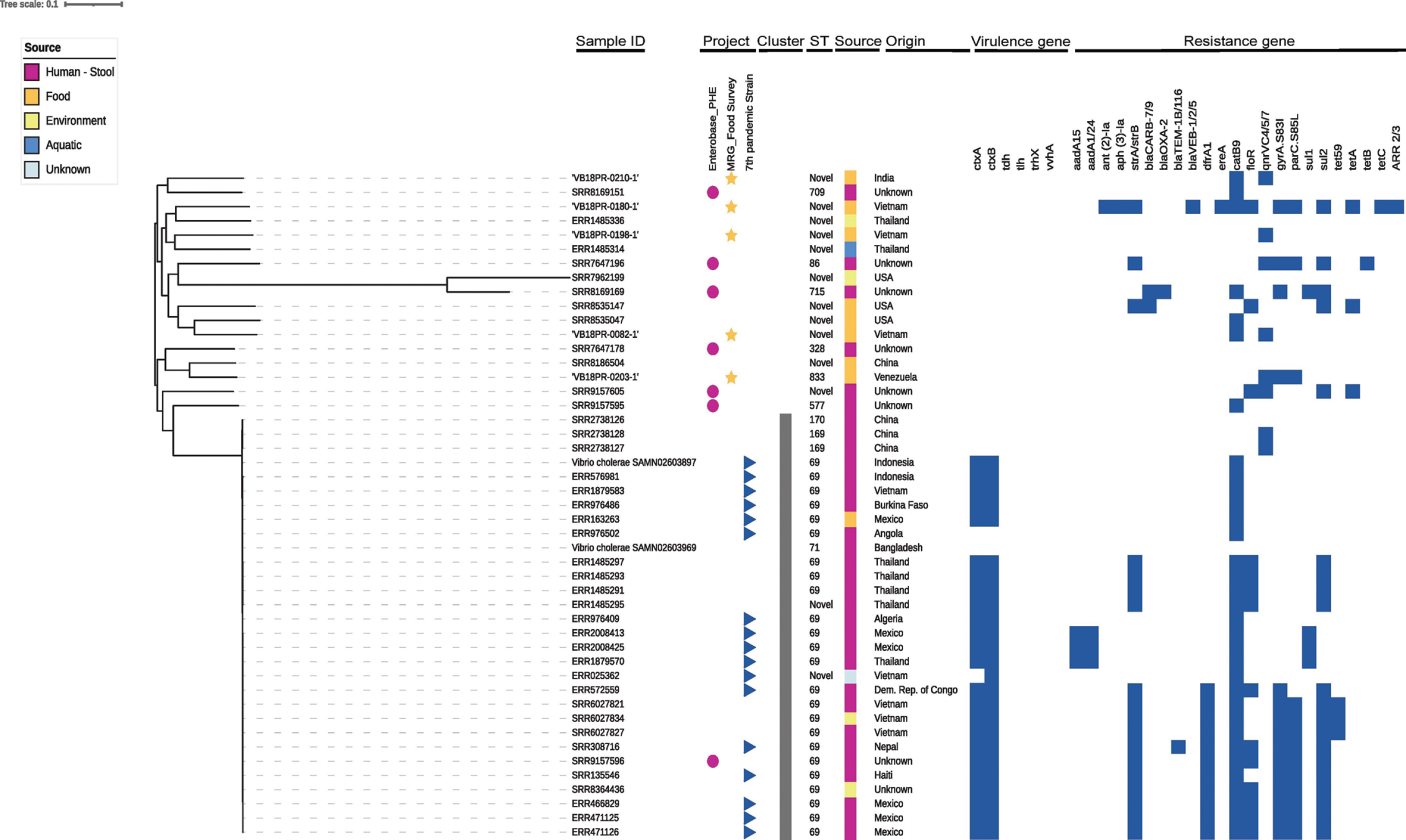
Maximum-likelihood phylogeny using core genome alignment of five *V. cholerae* isolates collected from prawns sold at retail with 42 context genomes comprised of representatives of seventh pandemic, UK clinical, environmental and food genomes. ST represents sequence types, legend describes context reference source, cluster, sample source and resistance gene presence indicated in dark blue. Phylogenetic tree is rooted to reference *V. parahaemolyticus* reference (SAMD00058707).

*Vibrio cholerae* were isolated from 2 % (5/211) of prawn samples collected, accounting for 4 % (5/130) of overall *Vibrio* isolates recovered (Table 1). The *V. cholerae* isolates were derived from raw full-body (3/5) and raw headless peeled (2/5) prawns. All samples containing *V. cholerae* originated from aquacultured sources with three samples originating from Vietnam, one from India and one from Venezuela (Fig. 1). These genomes were put in phylogenetic context with representative genomes of *V. cholerae* from the three waves of the seventh pandemic *V. cholerae* O1 El Tor, clinical isolates from patients in the UK, and isolates from other food, environment and clinical sources from various geographical regions (Fig. 3). Isolates from prawns collected in the current study did not cluster with any of the context isolates. The nearest clinical context genome had a pairwise non-recombinant SNP distance of 13 305 SNPs. MLST analysis resulted in four out of five prawn *V. cholerae* genomes identified as novel sequence types with one identified as ST833. No virulence genes associated with clinical cholera disease or known *Vibrio* associated human disease were found in food survey isolates. No putative plasmids were identified.

### Antimicrobial resistance

Whole genome sequence based *in silico* resistance gene detection identified one or more AMR genes in 77 % (100/130) of isolates from prawns and 12 % (15/130) of isolates contained genes associated with resistance to more than three antimicrobial classes. These MDR gene profiles were found in *V. parahaemolyticus *, *V. cholerae* and *V. vulnificus* (Table S12) and originated from Vietnam (13/15) and Thailand (2/15). Resistance genes were not detected in *V. alginolyticus *, *V. diabolicus *, *V. harveyi *, *V. mimicus* and *Vibrio* spp. (novel-1), and 5/25 of the remaining *Vibrio* isolates contained more than one resistance gene (Table S12).

**Fig. 4.**
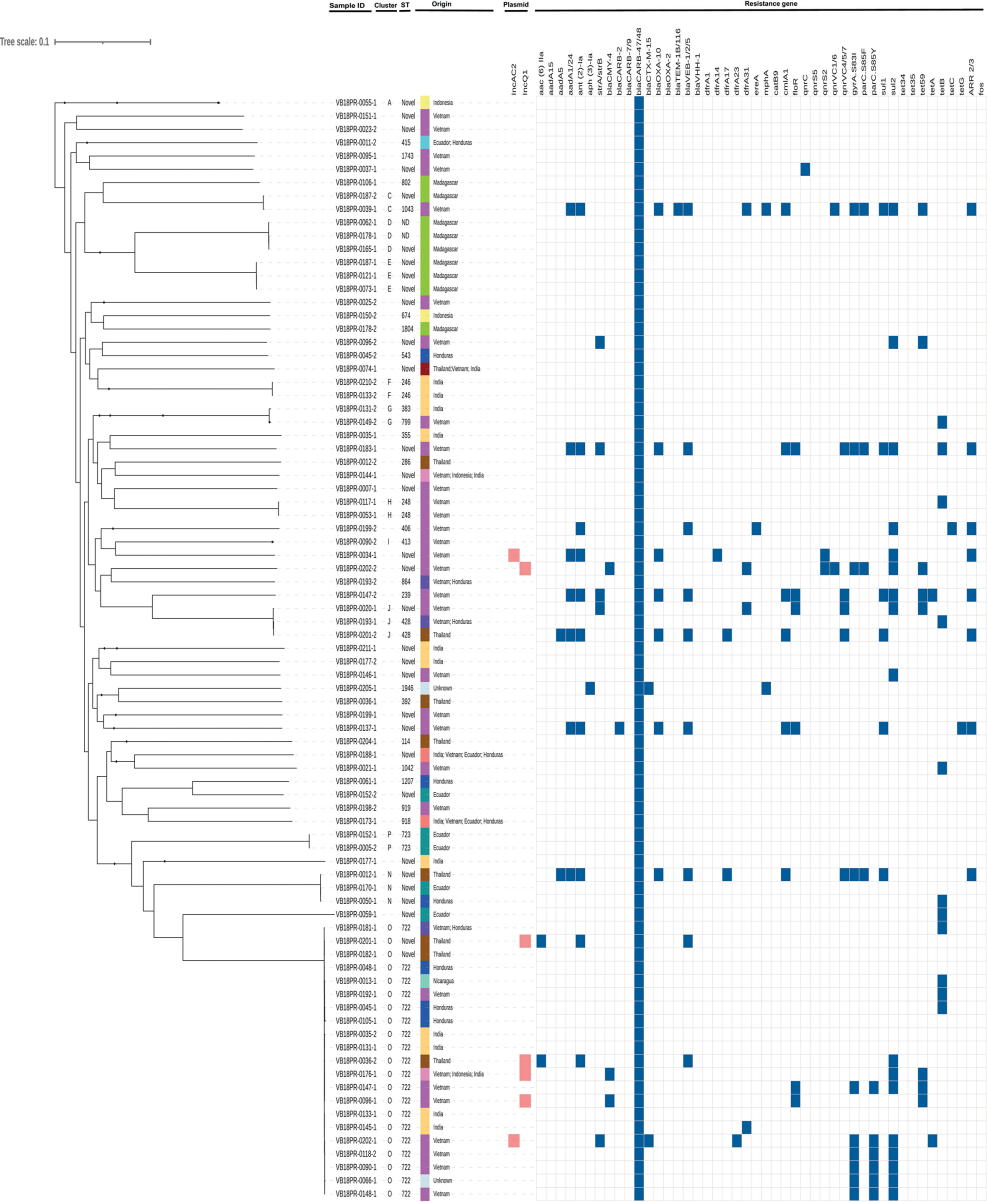
Maximum-likelihood phylogeny using core genome alignment of 83 *V. parahaemolyticus* isolates collected from prawns sold at retail. ST represents sequence types, colour strip with labels depicting country or combination of countries of origin, known plasmid replicons indicated in orange and resistance gene presence indicated in dark blue.

**Fig. 5.**
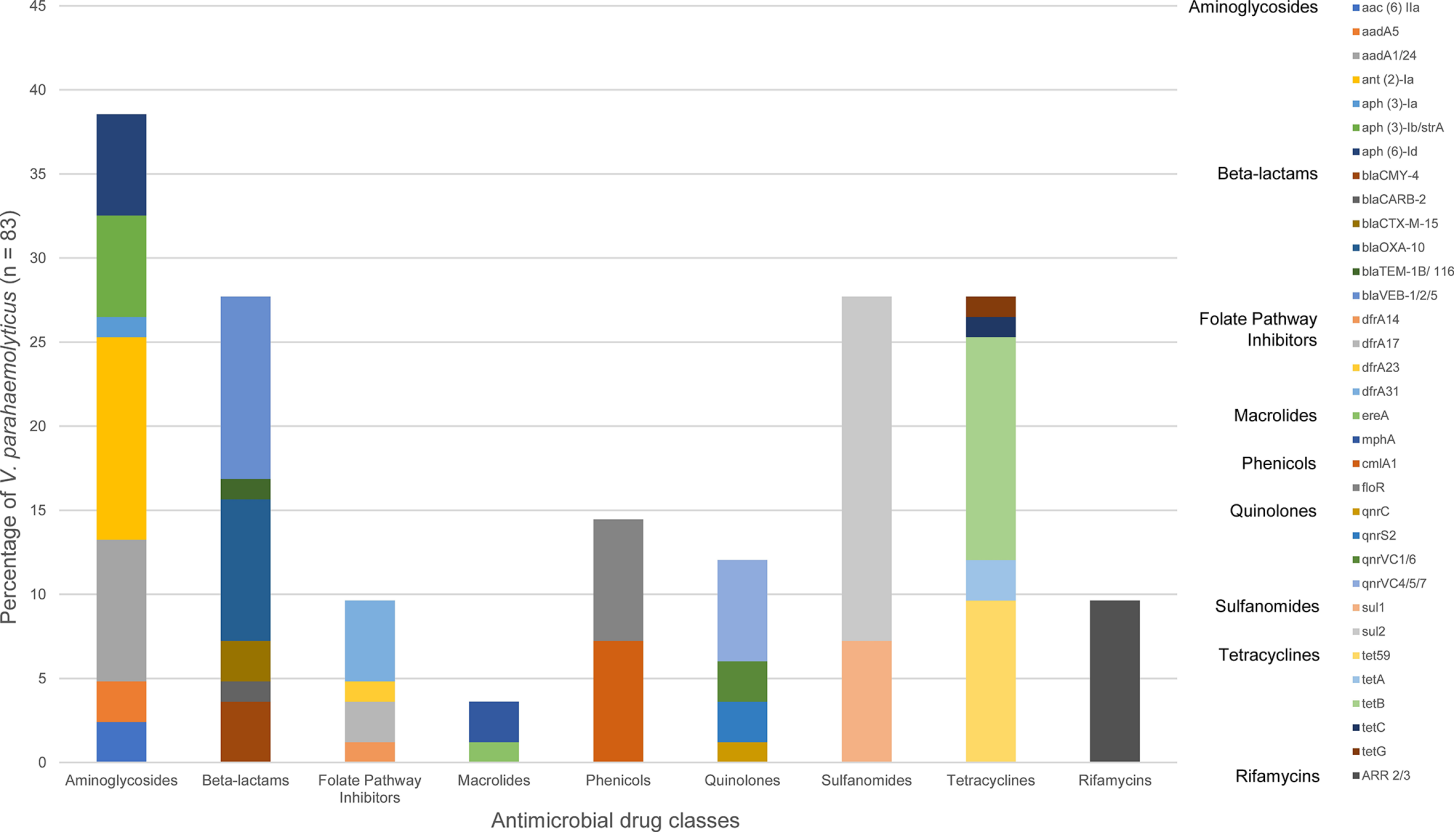
Distribution of antimicrobial resistance genes detected in *V. parahaemolyticus* isolated from prawns collected at retail by *in silico* WGS analysis.

Of the 83 *V. parahaemolyticus* genomes derived from prawns, all contained the intrinsic beta-lactamase gene *bla*CARB-47/48 and 42 % (35/83) contained one or more additional resistance gene (Fig. 4). MDR gene profiles were found in 20 % (17/83) of *V. parahaemolyticus *, significantly higher than in other *Vibrio* species (OR 3.74, *P*<0.05; CI 1.00, 21.12). The distribution of resistance genes within each class and the overall prevalence in all 130 food survey prawn isolates is shown in Fig. 5. Two isolates with MDR gene profiles carried extended spectrum beta-lactamase gene *bla*CTX-M-15. *V. cholerae* from prawns contained genes conferring resistance to one, two or seven classes of antimicrobials (Fig. 3). One isolate collected from aquacultured king prawns originating from Vietnam contained 13 resistance genes or SNPs conferring resistance to eight antimicrobial classes (Fig. 3).

### Intra-sample *Vibrio* variation

Intra-sample diversity was observed, as mentioned, with 13 samples containing *Vibrio* of different species and 20 samples containing two *Vibrio* of the same species. Of the 17 samples containing two *V. parahaemolyticus* isolates, there was significant diversity between the two isolates, with the isolates representing different sequence types, virulence genes and resistance gene profiles (Figs 1 and 2). The pairwise non-recombinant SNP differences between *V. parahaemolyticus* isolates from the same sample ranged from 1643 to 1977 SNPs. For samples with two different *Vibrio* species, 11/13 contained a combination with *V. parahaemolyticus* (Fig. 1).

The subset of three raw prawn samples each with six isolates selected demonstrates further evidence of intra-sample diversity. Two of the three samples contained six isolates each of *V. parahaemolyticus *, while one sample contained three *V. parahaemolyticus* and three *V. tubiashii* isolates. Diversity in the identities and numbers of AMR genes as well as plasmid identities was observed both within and between STs of *Vibrio* isolates found in the same sample (Table S9).

## Discussion

This study represents a comprehensive genomic investigation of predominantly non-cholera *Vibrio* isolates and provides insight into the diversity and epidemiology of food-derived *Vibrio *. With the high resolution provided by whole genome sequencing, it is possible to evaluate the degree of risk this food product represents to the consumer.

The twelve *Vibrio* species characterised in this study include the main four species (*V. parahaemolyticus, V. alginolyticus, V. vulnificus* and *V. cholerae *) associated with human foodborne and wound infections, as well as species responsible for marine animal infections. In this study, 46% of raw prawns available at retail were contaminated with at least one *Vibrio* species, and multiple isolates were obtained from 34 % of positive samples. Similar observations were described in Vietnam, where 56 % of prawns from retail markets contained multiple *Vibrio* species [59]. A mixed population of *Vibrio* within a single sample, either of multiple species or multiple strains of the same species, represents an opportunity for the transfer of genetic traits including virulence or toxin genes. Horizontal gene transfer (HGT) events have been described for various *Vibrio* species as a mechanism of adaption to changing environments [13, 60]. This includes the *tdh* gene within the pathogenicity island Vp-PAI in *V. parahaemolyticus* [60], and Type VI secretion system effectors (MIX-effectors) in *V. alginolyticus* [61]. In the changing environments from the aquaculture enclosures to the processing and packaging plants, vibrios from different natural niches mingle, compete and transfer functionalities, which could lead to acquisition of AMR or virulence traits resulting in adverse outcomes in the case of human infections.

This study has also demonstrated that the extent of product processing is associated with product contamination. The prevalence of *Vibrio* increased the more intact the exoskeleton was, i.e. shell-on prawns had a higher prevalence than peeled prawns, suggesting a critical point for contamination exposure which could be evaluated for pathogen control. The saline brine typically used in processing of prawns is conducive to the persistence of pathogens including *Vibrio* requiring salinity for survival throughout the food production chain [62, 63]. Of note, the majority of *Vibrio* isolated in this study were from raw prawns, and therefore cooking should remove any associated food safety risk. However, the presence of *Vibrio *, while not pathogenic, on a small number of cooked prawn samples suggests pathways exist that could allow pathogenic *Vibrio* species to contaminate these food products. The high prevalence of *Vibrio* in retail raw prawns has been evidenced at retail in southeast Asian countries [8, 24, 34, 64, 65]. Similar to this study, the most prevalent species identified in these countries was *V. parahaemolyticus *; in contrast, the prevalence of the non-*parahaemolyticus Vibrio* species was higher in other prawn and seafood studies than in this survey [8, 33]. This may be due to the wide variety of importing regions represented in the samples of this study, in comparison to the other studies which focussed on local seafood market samples within a single region or country.

The predominantly imported prawn products available in the UK retail market demonstrated the global nature of the UK food chain and reflect how environmental conditions of one region can have food safety implications worldwide.

Climate change, specifically the warming of water temperatures across all latitudes, sea level rise, changes in salinity, pH and nutrient availability have been identified as key factors to shifting *Vibrio* population structure and abundance in marine environments [9, 66–68]. It has been suggested that *Vibrio* is an ideal sentinel organism for monitoring climate change in marine environments due to the impact of severe weather events, precipitation and temperatures on pathogenicity, abundance and HGT events [9, 69]. Outbreaks of vibriosis are associated with heatwaves, warmer waters and extreme weather events [9], while a combination of laboratory analysis and prediction models indicate rising sea levels to increase *V. vulnificus* exposure to widespread populations [67]. The evidence of climate change on *Vibrio* in natural environments in recent years has been mounting, and the conclusions are that it will result in a negative impact on human health [9, 66, 68–70]. In addition, it is estimated that persons 65 years and over will double worldwide in 30 years [71]. This changing population of traditionally higher risk groups, coupled with increasing risks to the burden of illness due to climate change are predicted to increase the incidence of foodborne and waterborne pathogen infections, including disease causing *Vibrio* [9, 71, 72]. Although climate data were not taken into account in this study, it can be envisaged that future spill over of these changing *Vibrio* populations will be present in prawns available to consumers if no interventions are put in place along the production chain.

There is a lack of public health surveillance and food risk assessments for non-O1/139 *V. cholerae* and non-*cholerae* vibrios to understand fully the clinical impacts. To date, non-cholera vibrios are not a notifiable pathogen in the UK and food pathogen surveillance programmes do not actively test and analyse *Vibrio *. Until recently, the epidemiology of *V. parahaemolyticus* infections in the UK was relatively unknown. In a recent study focusing on the genomic epidemiology of clinical *V. parahaemolyticus* isolates collected in the UK study spanning a ten year period, most infections were related to recent travel to southeast Asia [2]. The retrospective study of UK-derived human *V. parahaemolyticus* infections found high genomic diversity, with ST3 predominantly responsible for the foreign-acquired infections [2]. In this current study, no sequence types associated with pandemic clone groups ST3 or ST36 were found. Although three prawn derived genomes from this study were phylogenetically close (9–26 non-recombinant pairwise SNP differences) with genomes obtained from clinical cases in the UK, the prawn and clinical genomes had different STs and different sets of plasmids, AMR and virulence genes. In addition, the isolates were collected in different years, suggesting that the human clinical cases had been acquired elsewhere.

The ubiquitous nature of *V. parahaemolyticus* in marine and estuarine environments explains its presence in prawns originating from all geographical regions in this study. The population structure of *V. parahaemolyticus* has previously been categorised into clearly differentiated geographical regions, but the movement of human activity has disrupted the continental zoning of the populations, thereby allowing opportunity for more genetic exchange [35]. In terms of a food safety risk, genes associated with disease (*tdh, trh*) were not found in *V. parahaemolyticus* in this study, suggesting prawns did not carry pathogenic strains. These two virulence genes in *V. parahaemolyticus* continue to be used in clinical, surveillance and research testing as an indicator of pathogenicity [9, 10, 24, 33, 73]. To a lesser extent, instances of vibriosis in patients have been caused by *tdh*-/*trh- V. parahaemolyticus*, as seen in the UK clinical isolates investigated in this study [73–75]. Although the *tdh* and *trh* virulence genes were not found in this study, there is limited understanding of other emerging virulence factors that are not yet well defined and may be present. Additionally, it is known HGT plays a role in pathogenicity transfer [60], therefore continued monitoring and investigation into other pathogenicity markers in these products is warranted.

In addition to *V. parahaemolyticus *, three other *Vibrio* species of human health relevance were detected in this study, albeit in low (<2 % of the total) numbers. *V. cholerae* isolates described in this study were distinct from all comparator pathogenic strains of *V. cholerae *, sharing no similarities with respect to virulence, ST or core genome clustering. To this effect, prawns sold at retail in the UK do not appear to be a vehicle of transmission for pathogenic or pandemic strains of *V. cholerae* causing cholera. Environmental sources of *V. cholerae* are associated with marine and estuarine ecologies as free-living bacteria or as part of microbiomes of particles or organisms, including seafood and have been linked to human gastrointestinal and wound infections [13, 54, 76, 77]. Surveys of sea water and seafood have observed higher levels of non-O1/O139 *V. cholerae* in comparison to this study [13, 33, 76, 78]. *Vibrio* are sensitive to climate conditions, therefore differing levels may in part be due to seasonality of harvest and changing climate condition, as seen in studies sampling at different time points [9, 13, 77, 79].

The antimicrobial classes of aminoglycosides, quinolones and many beta-lactams are listed as critically important for treatment in human medicine, while sulphonamides and tetracyclines are listed as highly important antimicrobials [80]. The agricultural use and/or resistance to these classes have been monitored in national surveillance systems such as CIPARS, NARMS in North America and ESVAC and EARS-Net in Europe, yet apart from the USA-based NARMS and COVIS, surveillance systems do not include non-cholera *Vibrio* in the AMR reporting and antimicrobial use reporting for aquaculture is limited [81–85]. Resistance determinants to critically important antimicrobial classes were identified predominantly in *V. parahaemolyticus* isolates, with over 50 % of isolates containing more than one AMR gene. The genes conferring resistance to aminoglycosides were found in 39 % of *V. parahaemolyticus *, with 28 % of isolates containing genes that conferred resistance to beta-lactams, sulphonamides and tetracyclines, respectively and 24 % of isolates contained quinolone resistance. Surveillance data capturing antimicrobial treatment regimens for *Vibrio* infections in the US indicate the use of quinolones, third and fourth generation cephalosporins, tetracyclines and penicillins [86]. Examining more than two decades of surveillance data, the study found that only quinolone use for treating vibriosis significantly decreased mortality, and for *V. vulnificus *, a combination of tetracycline, third generation cephalosporins along with quinolones was required to be effective in potentially lethal infections [86]. Of potential concern in this study was the 20 % prevalence of MDR gene profiles in *V. parahaemolyticus* and *V. cholerae *, particularly the seven different prawn samples containing MDR gene profiles with a combination of aminoglycoside, beta-lactam and quinolone resistance determinants. Samples with this MDR gene profile originated from Vietnam and Thailand. In an AMR prevalence study in Vietnam, 13.5 % of *Vibrio* expressed phenotypic MDR profiles [8]. This observation is supported by high levels of resistance observed in prawns purchased in Vietnam and the level of known antimicrobial use, residues on seafood products and resistance in the region [8, 19, 25, 26]. However, drug-resistant *Vibrio* from prawns have been observed in many parts of the world, as their farming practices are known to use high volumes of antimicrobials [19]. The majority of previous research on AMR in foodborne pathogens from prawns has used phenotypic methods, rather than whole genome sequencing; this study will provide a valuable resource for future work characterising the AMR burden in *Vibrio* in the food chain.

The *V. vulnificus* species consists of a wide range of strains that differ in pathogenicity. Multiple tests have been developed to distinguish between clinical- and environmental-associated strains. Two of the three *V. vulnificus* isolates in this study of retail prawns contained the *vcgC* allele, and all three contained the *pilF* polymorphisms, which Dickerson Jr. *et al.* found were associated with clinical cases rather than environmental water samples [15]. The *vvhA* gene is a virulence indicator that can induce autophagy-related cell death [87]. Although *V. vulnificus* isolates from retail prawns contained the *vvhA* gene, it is not clear what role this gene plays in human pathogenicity; one study found that *vvhA* knockouts did not have an altered virulence in mouse models, whereas another demonstrated that *vvhA* was cytotoxic in mice [12, 88]. A focused investigation of *V. vulnificus* isolates is warranted to further study virulence factors to assess the potential of retail prawns as a source of *V. vulnificus* infections.

One key finding of this study is the high diversity of *Vibrio* within a single sample. This has critical implications for source attribution and outbreak investigation; if only a single isolate is tested per sample, it is very possible that true transmission events will not be identified. In this study, *Vibrio* was isolated from prawn samples based on the ISO 21872-1 : 2017 methodology approved for use in food samples. Although all culture-based methods have limitations in that they may select for a subset of the organisms present, the selection of multiple *Vibrio* colony characteristics and multiple colonies per sample broadened the range of the *Vibrio* population identified in retail prawns. This study not only identified a wide range of species, but also diversity within a single sample and a putative novel *Vibrio* species. However, some *Vibrio* species may not be culturable using this method and therefore would not have contributed to this study. To address this, culture-free metagenome sequencing or the use of specific PCR primers could be used in the future to compare to the diversity of *Vibrio* obtained through culture. The application of whole genome sequencing and multiple isolates per sample in combination with robust epidemiological data in the food industry and public health surveillance will help identify sources of infection, track pathways of contamination and determine where food safety interventions could be most effectively applied.

Prawns are an important seafood commodity economically, as the UK imported 80 000 tonnes of prawns in 2018 at a value of over £640 million [22]. While acknowledging the limited surveillance of *Vibrio* in seafood, this study suggests that currently prawns are not a major source of *Vibrio* related infection in humans in the UK. However, the intensification of prawn production and increased consumption could lead to an expansion of *Vibrio* transmission through the global food supply chain [5, 8, 23]. As the climate continues to change, *Vibrio *-associated outbreaks in aquaculture stocks and the abundance of human pathogenic strains in subsequent prawn products are predicted to rise [9, 19, 22]. Since the UK imports the majority of prawns consumed, it is important to identify the overall level of contamination and population diversity of *Vibrio* present on raw prawns and the subsequent effort needed to strengthen food safety interventions throughout the production systems. However, the full extent of the public health impact cannot be determined without further surveillance of non-cholera *Vibrio* in foodborne infection cases and the food chain.

## Supplementary Data

Supplementary material 1

Supplementary material 2
