# Supplementary material for: Whole genome sequencing reveals great diversity of Vibrio spp in prawns at retail
Source: Microb Genom. 2021 Sep 28;7(9):000647. doi: 10.1099/mgen.0.000647 (PMC8715430; doi:10.1099/mgen.0.000647)
Supplement: Supplementary material 1 [file mgen-7-0647-s001.pdf]

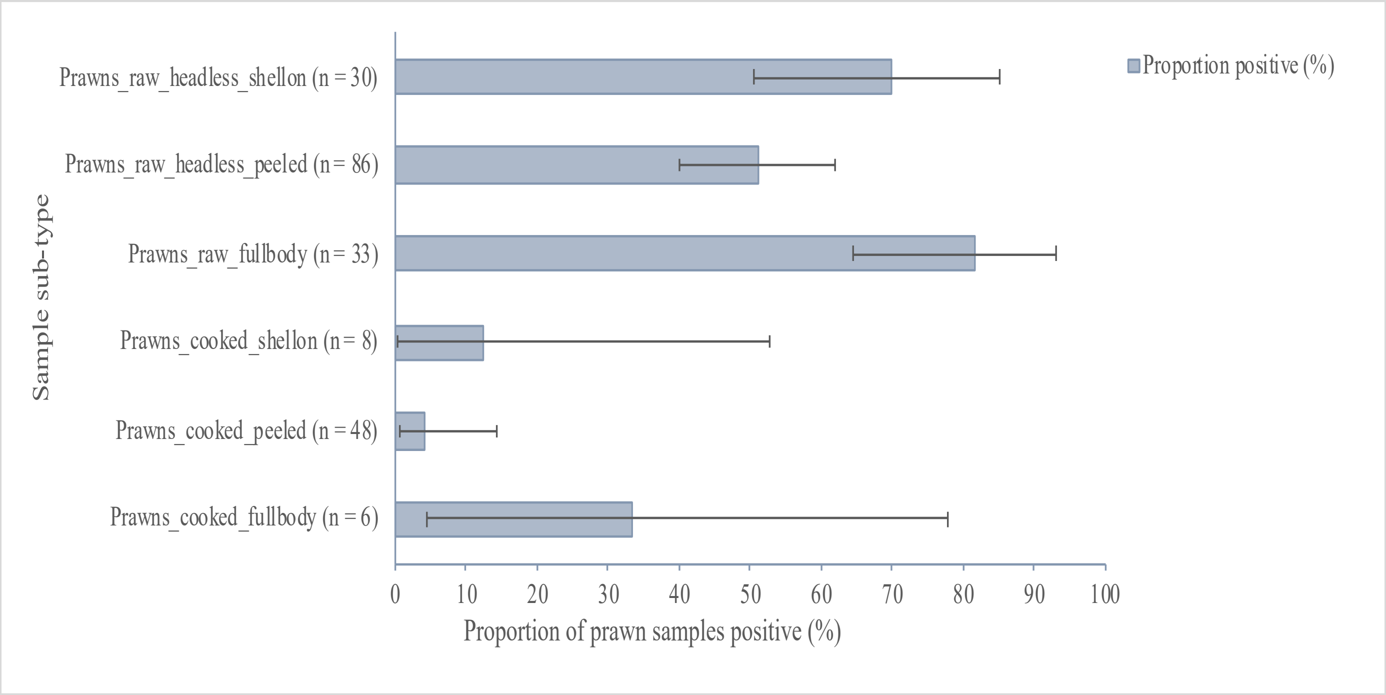

Supplementary figure 1. Proportion of prawn samples (n = 211) collected at retail testing positive for *Vibrio* spp.

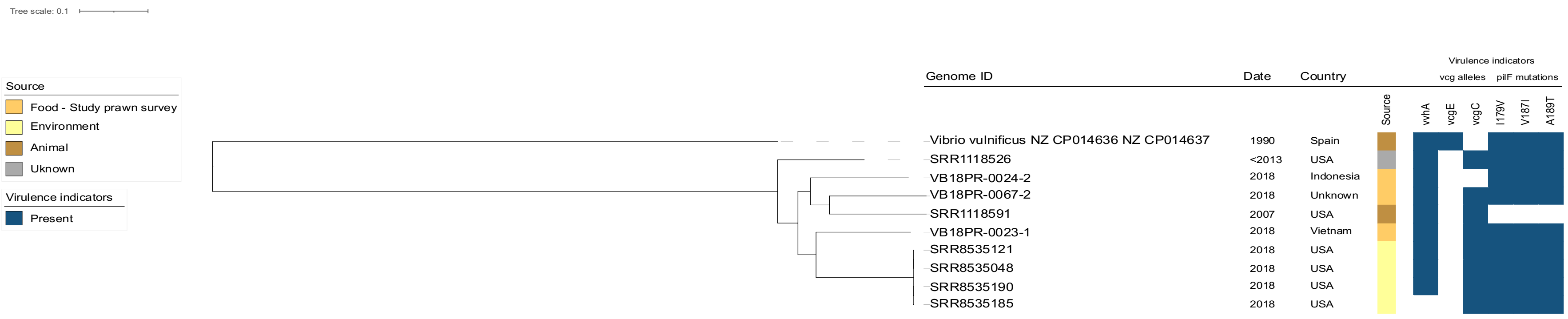

Supplementary figure 2. Maximum-likelihood phylogeny using core genome alignment of three *V. vulnificus* isolates collected from prawns sold at retail and seven context genomes of environmental and animal origin. Date of collection and country of sample isolation are labeled, with source depicted by a colour strip. Presence of virulence indidators are depicted in dark blue.

Supplementary table 1. Summary of population weighted study design and the number of sampling events in Norfolk, UK scheduled between May 2018 to April 2019.

Quartile	Population of Quartile	% of Norfolk	Strata	Population in Stratum	Stratum	Number of sampling events
Min	472		1			
1	28,984	4%	1	71,125	1	4
2	41,669	6%	1			
3	74,310	10%	2	74,310	2	4
4	396,978	54%	3	396,978	3	23
Max	186,682	25%	4	186,682	4	11

Supplementary table 7. Description of *Vibrio* species isolated from prawns by the region of product origin.

**Vibrio species (number of isolates)**	**Number (%) of Vibrio species**					
	**Southeast Asia[a]**	**India**	**Latin America[b]**	**Madagascar**	**Combination of Regions[c]**	**Other[d]**
**Samples tested (211)**	**75**	**20**	**32**	**12**	**34**	**38**
**Number of samples positive (97)**	**40**	**9**	**18**	**11**	**11**	**8**
**Number Vibrio isolates detected (130)**	**56**	**15**	**24**	**13**	**13**	**9**
V. parahaemolyticus (n = 83)	40 (47)	11 (13)	13 (15)	9 (11)	8 (9)	2 (2)
V. cholerae (n = 5)	3 (60)	1 (20)	1 (20)	0 (0)	0 (0)	0 (0)
V. alginolyticus (n = 4)	4 (100)	0 (0)	0 (0)	0 (0)	0 (0)	0 (0)
V. vulnificus (n = 3)	2 (67)	0 (0)	0 (0)	0 (0)	0 (0)	1 (33)
**Vibrio spp. (other) (n = 35)**	7 (21)	1 (3)	10 (30)	4 (12)	5 (15)	6 (18)
Vibrio owensii (n = 9)	2 (22)	0 (0)	3 (33)	3 (33)	1 (11)	0 (0)
Vibrio campbellii (n = 7)	0 (0)	0 (0)	5 (71)	1 (14)	1 (14)	0 (0)
Vibrio diabolicus (n = 6)	0 (0)	1 (17)	0 (0)	0 (0)	0 (0)	5 (83)
Vibrio tubiashii (n =6)	2 (33)	0 (0)	2(33)	0 (0)	1 (17)	1 (17)
Vibrio anguillarum (n = 3)	1 (33)	0 (0)	0 (0)	0 (0)	2 (67)	0 (0)
Vibrio harveyi (n = 1)	1 (100)	0 (0)	0 (0)	0 (0)	0 (0)	0 (0)
Vibrio mimicus (n = 1)	1 (100)	0 (0)	0(0)	0 (0)	0 (0)	0 (0)
Vibrio novel-1 (n=2)	0 (0)	2 (100)	0 (0)	0 (0)	0 (0)	0 (0)

[a] *Southeast Asia: Thailand, Vietnam, Indonesia*

[b] *Latin America: Ecuador, Honduras, Nicaragua, Venezuala, Angentina, combination of Latin American countries*

[c] *Combination of regions: India, Vietnam, Ecuador, Honduras; Thailand, Vietnam, Belize, Honduras; Thailand, Vietnam, India; Vietnam, Honduras; Vietnam, Indonesia, India*

[d] *Other: Greenland, United Kingdom, Unknown*

Supplementary table 8. Univariable models comparing region of origin of *V. parahaemolyticus* in prawns.

Sample type comparison	*n* [b]	OR [c]	95% CI [d]	*P*
Southeast Asia vs. India	69	**0.15**	**0.006 - 0.93**	**0.04**
Southeast Asia vs. Madagascar	67	0.89	0.21 - 3.27	0.88
Southeast Asia vs. Central America	81	1.17	0.44 - 3.11	0.74

Significant differences are highlighted in bold

[b] n = total number of samples in analysis

[c] OR = odds ratio

[d] CI = confidence intervals

Supplementary table 10. Description of pairwise non-recombinant single nucleotide polymorphism (SNP) differences, sequence types, virulence genes and resistance gene profiles in *V. parahaemolyticus* clusters containing clinical and prawn samples from the UK

Cluster	Genome ID	referent	SNP difference	ST	Source	Region of origin	Virulence genes	Resistance genes
A	VB18PR-0055-1	*	*	Novel	Food survey - prawn	Southeast Asia	*tlh*	*bla*CARB-47/48
	SRR3743888		9	614	Health Canada - food	Canada	*tlh*	*bla*CARB-47/48
	SRR7232600		26	749	PHE - clinical	Unknown	*tdh, tlh*	*bla*CARB-47/48
G	VB18PR-0131-2	*	*	383	Food survey - prawn	India	*tlh*	*bla*CARB-47/48
	SRR7232588		25	8	PHE - clinical	Unknown	*tdh, tlh*	*qnr*S, *bla*CARB-47/48
	VB18PR-0149-2		23	799	Food survey - prawn	Southeast Asia	*tlh*	*tetB*, *bla*CARB-47/48
I	VB18PR-0090-2	*	*	413	Food survey - prawn	Southeast Asia	*tlh*	*bla*CARB-47/48
	SRR7232606		22	1917	PHE - clinical	Unknown	*tlh*	*bla*CARB-47/48

Supplementary table 11. Number and description of identified clusters in the *V. parahaemolyticus* phylogeny

**Cluster**	**SNPs range**	**Number of genomes in cluster**
A*	0-26	**3**
B	0-11	**17**
C*	12	**2**
D*	0-1	**3**
E*	0-1	**3**
F*	3	**2**
G*	20-25	**3**
H*	0	**2**
I*	22	**2**
J*	3-7	**3**
K	3	**2**
L	0	**2**
M	7	2
N*	2-5	3
O*	0-18	21
P*	4	2

*indicates retail prawns included in cluster
